# *Drosophila melanogaster* as a model to study age and sex differences in brain injury and neurodegeneration after mild head trauma

**DOI:** 10.3389/fnins.2023.1150694

**Published:** 2023-04-03

**Authors:** Changtian Ye, Joseph A. Behnke, Katherine R. Hardin, James Q. Zheng

**Affiliations:** ^1^Department of Cell Biology, Emory University School of Medicine, Atlanta, GA, United States; ^2^Department of Neurology, Emory University School of Medicine, Atlanta, GA, United States; ^3^Center for Neurodegenerative Diseases, Emory University School of Medicine, Atlanta, GA, United States

**Keywords:** traumatic brain injury, neurodegeneration, sex difference, aging, risk factors

## Abstract

Repetitive physical insults to the head, including those that elicit mild traumatic brain injury (mTBI), are a known risk factor for a variety of neurodegenerative conditions including Alzheimer’s disease (AD), Parkinson’s disease (PD), and chronic traumatic encephalopathy (CTE). Although most individuals who sustain mTBI typically achieve a seemingly full recovery within a few weeks, a subset experience delayed-onset symptoms later in life. As most mTBI research has focused on the acute phase of injury, there is an incomplete understanding of mechanisms related to the late-life emergence of neurodegeneration after early exposure to mild head trauma. The recent adoption of *Drosophila*-based brain injury models provides several unique advantages over existing preclinical animal models, including a tractable framework amenable to high-throughput assays and short relative lifespan conducive to lifelong mechanistic investigation. The use of flies also provides an opportunity to investigate important risk factors associated with neurodegenerative conditions, specifically age and sex. In this review, we survey current literature that examines age and sex as contributing factors to head trauma-mediated neurodegeneration in humans and preclinical models, including mammalian and *Drosophila* models. We discuss similarities and disparities between human and fly in aging, sex differences, and pathophysiology. Finally, we highlight *Drosophila* as an effective tool for investigating mechanisms underlying head trauma-induced neurodegeneration and for identifying therapeutic targets for treatment and recovery.

## Introduction

Neurodegenerative diseases, such as Alzheimer’s disease (AD), Parkinson’s disease (PD), Huntington’s disease (HD), and frontotemporal dementia (FTD), involve progressive disruption of brain function and subsequent neuronal loss that are more prevalent in aged populations. While an increasing number of risk genes and mutations have been identified, these genetic factors only account for a small portion of neurodegenerative cases (Bertram and Tanzi, 2005; Hardy and Orr, 2006; Pihlstrøm et al., 2018; Fu and Ip, 2023). Aging is the main risk factor for many neurodegenerative disorders and accelerated unhealthy aging likely plays a key role in the development of neurodegenerative conditions (Azam et al., 2021; Saikumar and Bonini, 2021). Other significant risk factors include environmental insults such as exposure to trauma, drugs, or toxins which can have harsh consequences later in life, including the development of dementia (Brown et al., 2005; Gupta and Sen, 2016; Schaefers and Teuchert-Noodt, 2016). In fact, head trauma induced by physical impacts is one of the greatest external risks for brain injury and disability, often inflicting a heterogeneous set of physical, cognitive, and emotional symptoms affecting millions of individuals each year worldwide (Roozenbeek et al., 2013; Heinzelmann et al., 2014; Ling et al., 2015; Stein et al., 2015). In particular, closed-head traumatic brain injury (TBI) represents one of the most common head injuries associated with contact sports, automobile accidents, and falls. The majority of TBI cases can be classified as mild TBI (mTBI) as they involve limited loss of consciousness and memory without diagnosable structural brain alterations (CDC, 2003; Cassidy et al., 2004). Mild head trauma can cause long-term disability and/or lead to late-life brain degeneration, such as chronic traumatic encephalopathy (CTE), AD, and other progressive neurodegenerative conditions (Ojo et al., 2016; Asken et al., 2017; Mez et al., 2017; Graham and Sharp, 2019; Brett et al., 2022). Even sub-concussive head impacts, especially when exposed repetitively, can cause significant cognitive impairment later in life (Ntikas et al., 2022). Previous preclinical TBI studies have implicated the involvement of neuronal excitotoxicity, increases in intracellular calcium, free radical production, mitochondrial dysfunction, and inflammatory mediators (Globus et al., 1995; Werner and Engelhard, 2007; Hawryluk and Manley, 2015) within the injury response cascade, as well as the disruption of blood brain barrier integrity (Shlosberg et al., 2010), activation of glia (Myer et al., 2006), and inhibition of regeneration (McGraw et al., 2001; di Giovanni et al., 2005; Itoh et al., 2007). Within these pathways, mitochondrial dysfunction and chronic inflammation have been suggested to be involved in long-term processes leading to neurodegenerative conditions (Wang et al., 2019, 2020; Gao et al., 2022). However, the study of many of these processes in preclinical models of mTBI has been limited to the acute phase of injury and it remains to be determined if any of these processes are responsible for the emergence of neurodegeneration years after injury exposure.

Age and sex are two important biological factors that affect outcomes following mild head trauma. Both clinical and preclinical studies show that aging is associated with worse outcomes following injury (Cheng et al., 2014; Abdulle et al., 2018; Ritzel et al., 2019). Elderly individuals are more prone to developing severe and long-lasting behavioral symptoms than younger adults, but the underlying causes remain to be fully elucidated. Sex-related differences have been documented in many medical conditions, including neurodegenerative disorders (Li and Singh, 2014; Podcasy and Epperson, 2016; Au et al., 2017; Nebel et al., 2018; Cerri et al., 2019; Mauvais-Jarvis et al., 2020; Vegeto et al., 2020) and neurotrauma of various severities (Marar et al., 2012; Berz et al., 2013; Dollé et al., 2018; Gupte et al., 2019; Yue et al., 2019; Rauen et al., 2021). Sex differences and sex-related factors, including anatomical differences (Mansell et al., 2005; Dollé et al., 2018), gonadal hormone differences (Stein, 2001), reproductive status (Isaac et al., 2010; Garbe et al., 2016; Dove et al., 2017), and immunological responses (Ertürk et al., 2016; Jassam et al., 2017; Späni et al., 2018), presumably affect vulnerability to neurodegeneration. How sex-related factors impact short-term responses to mTBI and the subsequent development and progression of neurodegenerative conditions are poorly understood. Interestingly, aging progression is known to be different between male and female (Hagg and Jylhava, 2021), indicating the complex interplay between these two factors in neurodegeneration. Clearly, further development and utilization of preclinical animal models with short lifespans can greatly accelerate the research focused on the understanding of molecular and cellular mechanisms underlying sex differences and age-dependence in trauma-elicited neurodegenerative conditions that emerge late in life. In this review, we first outline the pathology of mTBI and subsequent neurodegenerative conditions. We then discuss current understanding of age and sex in the progression of trauma-mediated neurodegeneration from human and mammalian models. Finally, we highlight *Drosophila* models of mTBI and their potential in studying head trauma-induced neurodegeneration and the role of age and sex within the injury response.

## Traumatic brain injury and neurodegeneration

Traumatic brain injury is, “an alteration in brain function, or other evidence of brain pathology, caused by an external force” (Menon et al., 2010), that presents as a heterogeneous set of physical signs, and cognitive and emotional symptoms (Heinzelmann et al., 2014; Ling et al., 2015; Stein et al., 2015). The Centers for Disease Control and Prevention estimates an annual TBI incidence of 2.8 million individuals in the US, including 50,000 TBI-related fatalities (CDC, 2015). Over five million Americans are currently living with long-term TBI-related disability (CDC, 2015). Closed-head injuries, most commonly a result of falls and automobile accidents, are the most prevalent type of TBI, of which mild TBI, also known as *concussion*, account for ~75% of total TBI incidence (CDC, 2003). This is likely an underestimate as mTBI often goes undiagnosed, or even unrecognized in cases where medical care is not pursued. mTBI may present with subtle or even no obvious indications following injury, yet 11–38% of affected individuals experience symptoms 6-months after injury (Voormolen et al., 2018), indicating that mTBI may have long-lasting neurological effects (Nelson et al., 2019). Studies that compared asymptomatic athletes who play contact sports to athletes who play non-contact sports revealed long-term brain changes (Slobounov et al., 2017; Manning et al., 2019) and cognitive deficits (Killam et al., 2005; McAllister et al., 2012; Talavage et al., 2014), suggesting that even seemingly innocuous head impacts regardless of symptoms can lead to deleterious effects later in life. Furthermore, a history of repetitive mTBI, especially in high-risk individuals such as military personnel (Warden, 2006; Barnes et al., 2018) and contact athletes is associated with the insidious progressive neurodegenerative disease CTE, where symptom onset occurs years after exposure (Martland, 1928; Blennow et al., 2012; DeKosky et al., 2013; Baugh et al., 2014; Ling et al., 2015; Stein et al., 2015; McKee et al., 2016; Mez et al., 2017; Mackay et al., 2019). A prior history of head trauma is also associated with the development of age-related neurodegenerative diseases (Little et al., 2014; Esopenko and Levine, 2015; Perry et al., 2016; Asken et al., 2017; DeKosky and Asken, 2017; Mez et al., 2017; Erkkinen et al., 2018), including AD (Mortimer et al., 1991; Barnes et al., 2018; Katsumoto et al., 2019), PD (DeKosky et al., 2013), and amyotrophic lateral sclerosis (ALS; Little et al., 2014), which collectively represent a leading cause of long-term morbidity and mortality worldwide (Erkkinen et al., 2018). Despite the critical concern related to repetitive head trauma exposure, its underlying mechanisms leading to neurodegeneration and long-term complications remain poorly understood (Asken et al., 2017; Fehily and Fitzgerald, 2017; Wojnarowicz et al., 2017).

Traumatic brain injury can be broken down into two injury components that are sustained in tandem: the primary injury, which is the initial immediate mechanical impact/insult sustained by the brain that results from linear and/or rotational head movement, followed by the secondary injury, which involves the subsequent myriad of downstream pathophysiological processes (Werner and Engelhard, 2007). Secondary injury consists of excitotoxicity, free radical production, mitochondrial dysfunction, and inflammatory mediators (Werner and Engelhard, 2007; Hawryluk and Manley, 2015) and can sustain for days, weeks, months and even longer depending on the nature of the injury. Axons are thought to be the most vulnerable portion of the neuron to mechanical trauma, given their large surface area to volume ratio (McKee and Daneshvar, 2015) and high degree of anisotropic organization of cytoskeletal elements (Johnson et al., 2013), principally composed of microtubules (Conde and Caceres, 2009). Shear stress and strain forces elicited by mechanical head trauma can cause rapid stretching of axons, followed by an unregulated influx of cations (Na^+^ and Ca^2+^) through sodium and calcium channels along the axolemma, resulting in indiscriminate depolarization and release of excitatory neurotransmitters (Lyeth et al., 1993; Palmer et al., 1993; Globus et al., 1995; Wolf et al., 2001; Yi and Hazell, 2006; Weber, 2012; Fehily and Fitzgerald, 2017). Persistent neuronal hyperexcitability can overburden Na^+^/K^+^ pumps needed to restore proper ionic homeostasis, and can last into the chronic phase of injury (Lima et al., 2008). Elevated ion pump activity can also overwhelm mitochondrial buffering of calcium and eventually result in metabolic dysfunction (Barkhoudarian et al., 2016). In addition to ionic disturbances, structural abnormalities are seen immediately following brain injury, including the breaking of microtubules directly in response to physical trauma (Tang-Schomer et al., 2010). Concurrently, elevated calcium can activate calcium-sensitive proteases, such as calpain, which cleave cytoskeletal components, such as microtubules within the axon (Saatman et al., 1996a,b, 1998). Microtubule degradation is evident in other parts of the neuron as well. Hippocampal microtubule-associated protein 2 (MAP2), which is localized mainly to the soma and dendrite, is also reduced following injury (Taft et al., 1992). Inhibiting calpain activation shortly after injury mitigates behavioral deficits and cytoskeletal breakdown in preclinical animal models of injury (Saatman et al., 1996b; Posmantur et al., 1997). Subsequent processes include axonal transport deficits (Ma et al., 2016), swelling (Smith et al., 2003), retraction (Johnson et al., 2013), cellular inflammation (Giza and Hovda, 2014) and eventual cell death (Giza and Hovda, 2014). The culmination of these processes is theorized to give rise to neurodegeneration, such as CTE pathology found in post-mortem brains from individuals with a history of repetitive trauma (McKee et al., 2016). CTE pathology is characterized by the presence of hyperphosphorylated tau accumulated within neurons and glia surrounding small blood vessels (perivascular) at sulci depths in a pattern distinct from AD (McKee et al., 2016; Mez et al., 2017). Other supportive pathological features include abnormal TDP-43 accumulation (McKee et al., 2013, 2016; Mez et al., 2017), and may include beta-amyloid (Aβ) deposition (McKee et al., 2016). The subsequent accumulation of toxic protein aggregates is thought to result in chronic neurodegeneration (Little et al., 2014; DeKosky and Asken, 2017; Gilmore et al., 2020). In addition to parenchymal brain loss, neurodegeneration is seen in other nerve fibers, including the progressive response that is evident in retired veterans who experience a decrease in longitudinal retinal nerve fiber layer (RNFL) thickness (Gilmore et al., 2020).

The mechanisms that connect primary and secondary injury sequelae to long-term neurodegeneration remain undefined. It is also unclear whether concussion and sub-concussive injuries represent distinct pathophysiological processes. Given that even mild injury exposure carries long-term risks, it is imperative to understand the injury cascades elicited during the latent or asymptomatic stage of injury that may potentiate late-life neurodegeneration. To date, there are no FDA-approved drugs explicitly developed for treating mTBI. This dearth of therapeutics leaves the treatment of moderate and severe cases of trauma with limited options to promote functional recovery aside from supportive and life-saving hemodynamic strategies. With limited therapeutics at our disposal for the treatment of head trauma, the identification of risk factors associated with worse outcomes following mild head trauma is an important component for mitigating risk in vulnerable populations. Here, we will discuss biological sex and age as two important risk factors in mTBI and long-term brain deficits.

## Age affects mTBI outcome and subsequent neurodegeneration

Age is one of the strongest outcome predictors for complications following head trauma, including mild trauma (Jacobs et al., 2010; Moretti et al., 2012; Bittencourt-Villalpando et al., 2020). The cause of injury varies by age, with motor vehicle accidents being the greatest source of injury in young adults and falls being the greatest contributor to injury in elderly populations (Peterson et al., 2019). Although the cause of injury differs, older age is consistently associated with an increased incidence of head trauma, a slower overall recovery process, as well as greater morbidity and mortality following injury (Abdulle et al., 2018). Compared to the high rates of head trauma in the elderly, studies on the intersection of aging and mTBI-related neurodegeneration are surprisingly scarce. Functional outcome 6-months following injury reveals a slower recovery in affected older individuals (aged 60+ years of age) who initially present with the same degree of mild injury severity compared to younger adults (Mosenthal et al., 2004). Another study with mildly injured individuals 65+ years of age revealed that the level of one’s education positively affected the chances for reaching a full recovery 6-months following injury (van der Naalt et al., 2017). Together, these findings lend support to the concept of cognitive reserve, which decreases with age and is associated with the onset of age-related neurodegenerative disorders such as AD (Stern, 2012), as a potential protective factor against the effects of mild head trauma. This is further supported by the finding that cognitive reserve is associated with improvements in multiple tested cognitive domains (memory, verbal fluency, and executive function) across all injury severities of head trauma 1-year following injury in an adult cohort ranging from 19 to 79 years of age (Steward et al., 2018). At the cellular level, aging neurons in both the central and peripheral nervous system lose regenerative potential (Verdú et al., 2000; Nicaise et al., 2020), which may contribute to the increased vulnerability of aged individuals to developing brain deficits after exposure to mTBI. At this moment, it is difficult to disentangle the added effect of comorbidities and increasing frailty associated with aging, but pre-clinical work has begun dissecting these potential mechanisms which will then inform further clinical investigation.

There is generally an underrepresentation of young and aged cohorts in mTBI studies, accounting for less than 5% of rodent mTBI studies (Bodnar et al., 2019). Models of mTBI which more closely mimic clinical conditions, such as closed-head injuries, are very rarely conducted in aged animals (Iboaya et al., 2019). This is not to say that there is no evidence of the effects of age on mTBI-induced changes in the brain. Two studies have highlighted that age-at-injury affects long-term behavioral outcomes (Rowe et al., 2016) and neuropathology (Doust et al., 2021). Both studies subjected male rats to a single mild fluid percussion injury at post-natal day 17, day 35, 2-, 4-, or 6-months-old, the oldest time point roughly corresponding to a young adult human. Rats injured at earlier developmental timepoints were more vulnerable to developing motor and cognitive deficits shortly after injury. Rats injured in adulthood showed increased anxiety-like behavior compared to sham controls. However, when assessed at 10 months of age, injured rats seemed to have recovered and behave like naïve control rats (Rowe et al., 2016). This behavioral recovery did not extend to neuropathology, as immunohistochemistry revealed dendritic and axonal damage as well as glial activation in specific regions of the brain, regardless of age-at-injury (Doust et al., 2021). Furthermore, rats injured in adulthood had greater dendritic neuropathology in cortical grey matter and higher extent of microglial activation in the hippocampus. Though further chronic timepoints were not examined, it is surmisable that continuous and irreversible damage caused by the mild injury that is already visible a few months following injury, accelerates neurodegeneration. Another study gave 18-month-old human tau transgenic mice a single or repeated (five impacts over 9 days) closed-head mild injury that did not cause direct tissue damage (Ojo et al., 2013). Three weeks after the injuries, the study found a significant increase in phosphorylated tau in the repeated injury group when compared to the sham and single injury group, and notably consistent astrocyte and microglial activation in the injured regions. This study suggests that repetitive injury augments already-present neurodegenerative tau pathology in aged animals. However, the study did not compare the aged cohort to a younger cohort, so it is unclear whether tau abnormalities in young adult mice affect the injury response and whether aging mechanisms interact with injury mechanisms.

What are the specific mechanisms of aging that interact and compound with mechanisms of head injury? How may that inform future treatment options targeting head injury-induced neurodegeneration in the vulnerable aging population? Research in aging revealed that mitochondrial function (Cui et al., 2012; Chistiakov et al., 2014) and immune function (Weyand and Goronzy, 2016; Haynes, 2020) decline with aging. Given that mitochondrial dysfunction (Cheng et al., 2012; Fischer et al., 2016; Kim et al., 2017) and compromised immune responses (Needham et al., 2019; Verboon et al., 2021) have been implicated in TBI-induced brain dysfunction and degeneration, they may underlie or contribute to aging-increased vulnerability to brain injury and development of neurodegeneration in response to head trauma. Nonetheless, current limitations in human and rodent studies include small sample sizes and inadequate sampling, long experimental timelines, and heterogeneity of behavioral pathology that may result from different experimental manipulations. As we will discuss later, fruit flies, with their relatively simple nervous system and short lifespan, are uniquely poised to answer these questions.

## Sex differences in mTBI and neurodegeneration

Sex-related differences have been documented in many medical conditions, including neurodegenerative disorders (Li and Singh, 2014; Podcasy and Epperson, 2016; Au et al., 2017; Nebel et al., 2018; Cerri et al., 2019; Mauvais-Jarvis et al., 2020; Vegeto et al., 2020) and neurotrauma of various severities (Marar et al., 2012; Berz et al., 2013; Dollé et al., 2018; Gupte et al., 2019; Yue et al., 2019; Rauen et al., 2021). The use of both sexes within preclinical TBI research has garnered greater attention recently, following a period of time that incorporated limited female representation in studies (Späni et al., 2018), and has become a greater point of emphasis since the NIH mandated that sex be considered a biological variable in 2016 (Späni et al., 2018; Woitowich and Woodruff, 2019). A growing body of evidence demonstrates that there exists a sex-dependent effect related to incidence and recovery from neurotrauma (Marar et al., 2012; Berz et al., 2013; Dollé et al., 2018; Yue et al., 2019; Rauen et al., 2021). This should come as little surprise given that there are sex differences in anatomy (Mansell et al., 2005; Dollé et al., 2018), sex gonadal hormones (Stein, 2001), and immunological responses (Ertürk et al., 2016; Jassam et al., 2017; Späni et al., 2018), which presumably all affect vulnerability to injury. There is an interesting disparity between clinical and pre-clinical sex-dependent findings, where female sex is associated with worse outcomes in human studies and better outcomes in preclinical studies (Gupte et al., 2019). Part of this disparity in findings is attributed to differences in injury severity and animal model (Gupte et al., 2019).

In a large literature review, human studies involving mild brain injury showed that the female sex was associated with worse outcome measures (Gupte et al., 2019). Human data from the TRACK-TBI study demonstrated that the female sex was associated with decreased six-month functional outcome measured using the Glasgow Outcome Scale-Extended (GOSE) following mild TBI (Yue et al., 2019). A recent cross-sectional human study revealed that females were more likely to report a less favorable health-related quality of life (HRQoL) during the chronic stage of mild TBI (10 years post-injury; Rauen et al., 2021). Additional evidence demonstrated that young female athletes may take longer to become symptom free following sports-related concussion (Berz et al., 2013). In a study conducted in 10-35-year-old patients, females report more sleep disturbances only after a single concussion (Oyegbile et al., 2017). In patients that reported more than one incidence of injury, there were no major differences. When the data was stratified by age, the study found sex differences in sleep disturbances but only in post-pubescent ages (>15), suggesting possible hormone interactions. It should be emphasized that women remain significantly underrepresented within sport and exercise science research (Cowley et al., 2021). The sex data gap needs to be addressed by future studies that focus on the gender and sex differences in the risk and outcome of brain injury of various severities and the subsequent treatment and care.

Preclinical studies that consider sex as a variable while studying mild head trauma reliably find trends of differing trauma responses between sexes. A recent study using a mild closed head injury model in adult rats found that females showed greater deficits in recovery and locomotive behaviors after either repetitive sub-concussive impacts or one single concussive impact, whereas males exhibited increased anxiety and depressive-like behaviors (Wilson et al., 2023). Similarly, another study found worse recovery and higher deficits in behavioral tasks such as spatial memory in female rats (Wirth et al., 2017). However, in more severe injury models, findings are less consistent. Many studies find that female animals exhibit more favorable outcomes than males (O'Connor et al., 2003; Shahrokhi et al., 2010, 2012; Sarkaki et al., 2013), suggesting that female sex hormones are neuroprotective and anti-inflammatory, while others find female animals exhibit greater deficits and neurodegeneration or have produced mixed results (Mollayeva et al., 2018). Detailed discussion on sex differences in TBI and the contributing factors can be found in several reviews (Gupte et al., 2019; Ma et al., 2019; Rubin and Lipton, 2019; Chaychi et al., 2022). It is important to note that preclinical data showing the better outcomes of the female sex in severe injury models were the foundation for testing the neuroprotective effects of progesterone in the ProTECT (Progesterone for Traumatic Brain Injury, Experimental Clinical Treatment) human clinical trials for severe neurotrauma (Wright et al., 2014). Despite preclinical evidence demonstrating neuroprotection, exogenous progesterone failed to meet the clinical end point (a 10% improvement in the Extended Glasgow Outcome Scale 6-months post-injury; Wright et al., 2014). The paradoxical finding that exogenous progesterone exhibits neuroprotective properties in preclinical models, yet females experience a worse prognosis may be attributed to the cyclical nature of progesterone, and forms the basis of what is known as the “withdrawal hypothesis” (Wunderle et al., 2014; Valera et al., 2021). The “withdrawal hypothesis” posits that the relative abundance of progesterone dictates injury vulnerability. Females who sustain a mild TBI during the luteal phase of menstruation (when progesterone is high) report a lower quality of life (EuroQoL/EQ5D) and worse self-reported outcome measures (Rivermead Post Concussion Questionnaire) 1 month following injury compared to injured females on birth control, who exhibit elevated levels of progestins (Wunderle et al., 2014). A similar trend is seen across age-groups within females: pre-menarche and post-menopausal women report lower 3-month post-concussive symptoms compared to women of child-bearing years (Bazarian et al., 2010), indicating that injury vulnerability may be related to the disruption or natural cycling of female gonadal hormones. Taken together, both clinical and preclinical data reveal sex differences in the response to TBI of varying severities, the recovery, and short- and long-term outcomes. These complex and often conflicting outcomes associated with different sexes further emphasize the need to include both sexes in both clinical and preclinical studies and demand better sampling and controlling of experimental subjects for hormonal cycles, aging, and social/psychiatric factors.

While the mechanisms underlying the sex differences in brain injury responses and development of neurodegenerative conditions remain to be elucidated, alterations in mitochondrial functions and immune responses are considered the key candidates. Mitochondria dysfunction is thought to be involved in the pathogenesis of many neurodegenerative diseases (Lin and Beal, 2006; Wang et al., 2019, 2020; Gao et al., 2022). Mitochondria play an important role in cell death *via* their release of pro-apoptotic factors after undergoing outer membrane permeabilization, which commonly occurs after TBI (Cheng et al., 2012; Fischer et al., 2016). Sex differences exhibited in mitochondrial function could potentially result in disparate sex responses to TBI. Females of reproductive age typically display better mitochondrial function, lower levels of reactive oxygen species, and have higher levels of antioxidant enzymes compared to males (Silaidos et al., 2018). This trend is reversed however when females undergo menopause, which could potentially contribute to the higher rates of AD observed in females (Silaidos et al., 2018) as well as post-TBI brain degeneration.

Sex differences in the immune system likely affects neuroinflammation and neurodegeneration after mTBI, which has been reviewed in great detail by Klein and Flanagan (2016). Females mount higher innate and adaptive responses across all species, which is believed to be somewhat of an evolutionary tradeoff between survival strategies and reproduction. Most sex differences in the innate immune response, such as expression and activation of Toll-like receptors, are encoded in the germline and directly result from sex chromosomes. Though not very well understood, sex differences in microglia may play a role in a variety of neurodegenerative conditions (Lopez-Lee et al., 2022). Microglia are the main players in the innate immune response in the brain (Mason and McGavern, 2022). Following brain injury, physical damage to the blood–brain barrier and astrocytes elicits microglial activation for repair. However, microglia may fail to return to a naïve state, particularly when microglia are continuously activated by repeated injuries, which can lead to aberrant deposition of proteins such as Tau. The adaptive immune response, such as T and B cell activity and infiltrating myelomonocytic cells, is also stronger in females regardless of age and may be involved in both acute and chronic stages of TBI (Klein and Flanagan, 2016). Sex hormones are believed to affect neurodegeneration by their regulation of immune responses (Mollayeva et al., 2021), and environmental factors such as nutrition or microbiome can also affect immunity. Finally, the age and reproductive status of an individual are also important determinants of sex differences in immune responses. It is clear that animal models with short lifespan, available molecular and genetic toolboxes, and better control of sex-related changes are needed to detangle the complex interplays of age- and sex-related differences and to elucidate the molecular, genetic, and cellular mechanisms underlying age- and sex-dependent late-life development of neurodegenerative conditions after environmental insults. As we discuss in the next section, fruit flies represent one of such animal models.

## *Drosophila melanogaster* as a model for studying neurodegenerative diseases and TBI

*Drosophila* have served as a powerfully tractable model organism to investigate fundamental neurobiological processes and mechanisms of neurodegeneration (Wittmann et al., 2001; Dias-Santagata et al., 2007; Chatterjee et al., 2009; Ali et al., 2011; Ling and Salvaterra, 2011; Burnouf et al., 2016; Sunderhaus and Kretzschmar, 2016; Coelho et al., 2018). This is made possible due to conserved neurobiology that exists between both *Drosophila* and mammalian species, including Na^+^/K^+^-based action potentials and inhibitory and excitatory neurotransmitters with shared neurosecretory-released mechanisms (Freeman, 2015). Like that of the mammalian brain, the fly brain consists of an organized arrangement of discrete neuronal structures and circuitry, but exists within a much smaller, more easily dissectible brain size that enables the study of individual neurons and their corresponding functional roles (Scheffer et al., 2020). The brain of *Drosophila melanogaster* is comprised of ~100,000 neurons (Kremer et al., 2017; Scheffer et al., 2020) and 10,000 glia (Freeman, 2015; Kremer et al., 2017), which makes it several orders of magnitude smaller than the human brain [86 billion neurons (Scheffer et al., 2020), with an equal number of glia (von Bartheld et al., 2016)]. Specifically, the fly brain consists of a central brain and two large optic lobes, each with an outer cortex that contains cell bodies and a synaptically-dense inner neuropil (Buchanan and Benzer, 1993; Ito et al., 2014). This is in contrast to the vertebrate brain, which features a synaptically-dense outer region within the superficial cortical layers (Subramanian et al., 2020). The outer cortex of the fly brain is surrounded by a perineurium that serves a functional equivalent to the blood–brain barrier within vertebrates (Buchanan and Benzer, 1993). The ventral nerve cord is the invertebrate equivalent of the mammalian spinal cord, which extends into the thorax where it relays motor-sensory information (Court et al., 2020). At the cellular level, *Drosophila* neurons share the same three subcellular compartments as mammalian neurons (axon, soma, dendrite), which make up a mix of unipolar and multipolar neurons (Rolls, 2011). However, unlike mammalian dendrites, *Drosophila* dendrites do not possess clearly demarcated spines that occupy post-synaptic sites (Rolls, 2011). Furthermore, the mammalian brain is a highly vascularized structure that is suspended and cushioned in cerebral spinal fluid (CSF), and contained within a boney skull, whereas the *Drosophila* brain is surrounded by trachea and air sacs that distribute nutrients and oxygen like the mammalian circulatory system and is enclosed within a chitinous exoskeleton. Although the *Drosophila* brain is not surrounded by CSF, the surrounding air sacs serve as a fluidic equivalent in being able to suspend the brain and serve as a buffer between the brain and outer enclosure.

**Figure 1:**
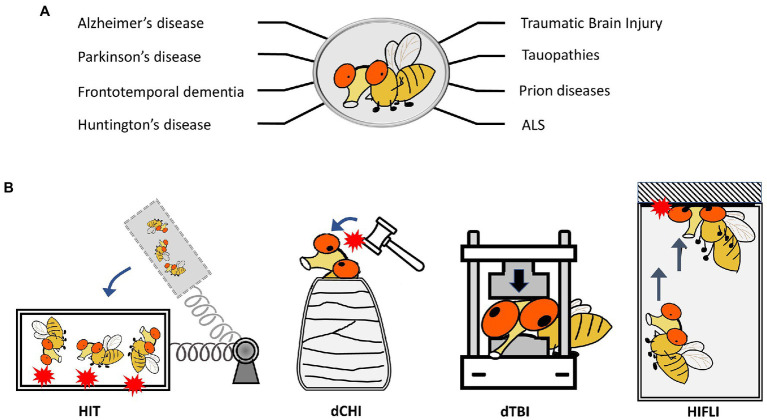
*Drosophila melanogaster* as a model to study neurodegenerative diseases and TBI. **(A)** Representative neurodegenerative diseases modeled in *Drosophila*. **(B)** Schematics showing four *Drosophila* models for TBI-related research. HIT: high impact trauma model by Katzenberger et al. (2013). dCHI: *Drosophila* controlled head-impact model by van Alphen et al. (2022). dTBI: *Drosophila* TBI model using a Piezoelectric actuator by Saikumar et al. (2020). HIFLI: Headfirst Impact FLy Injury model by Behnke et al. (2021).

Several key characteristics of *Drosophila melanogaster* make it an ideal model organism for the study of neurological diseases. 75% of disease-related genes in humans have corresponding fly orthologs (Pandey and Nichols, 2011). They have a short reproductive and life cycle, which enables lifelong processes such as those related to neurodegeneration and aging in a much more considerable time frame (Wittmann et al., 2001; Duffy, 2002; Jackson et al., 2002; Pandey and Nichols, 2011; Wang and Jin, 2011; He and Jasper, 2014; Coelho et al., 2018). Adult flies exhibit complex behaviors stemming from their organized central nervous systems, all while sharing conserved neural mechanisms with that of humans (Pandey and Nichols, 2011). Like humans, wildtype flies exhibit neurodegeneration associated with age. Reliable histological and behavioral assays have been developed to investigate aspects of neurodegeneration, such as examination of eye and retinal structures, scoring of brain vacuolization and abnormal protein deposits *via* immunohistochemistry, analyses of lifespan, measuring of sensorimotor functions using negative geotaxis assays (NGA), and assessment of the neuromuscular junction for synaptic phenotypes. Perhaps most impressive of the inherent *Drosophila* toolbox is the genetically tractable nature of fruit flies, which is unparalleled by any mammalian system. Many transgenic models of specific neurodegenerative diseases have been generated to study the progression of neurodegeneration, such as AD (Prüßing et al., 2013), PD (Whitworth, 2011), FTD (West, 2015), HD (Krench and Littleton, 2013), ALS (Casci and Pandey, 2015), and CTE (Aggarwal et al., 2022; Figure 1A). The ability to systematically probe protein function and control transgene expression in a cell-specific manner enables interrogation of various signaling mechanisms and opportunities for high-throughput screens. This approach has led to the study of cellular and molecular injury responses following axonal injury within the fly. Using a variety of genetic screens inherently accessible in *Drosophila* has led to the identification of several key genes and their respective proteins that are involved in axonal survival and degeneration (Collins et al., 2006; Xiong et al., 2010; Neukomm et al., 2014; Brace and DiAntonio, 2017); this includes Wallenda (Wnd), a fly homologue of dual leucine zipper kinase (a conserved mitogen-activated protein kinase (MAPK) important in cell-autonomous axonal degeneration following axonal transection). Importantly, these discoveries have translated to both murine (Osterloh et al., 2012; Shin et al., 2012; Watkins et al., 2013) and human cell-based (Tian et al., 2019) studies, which validate the utility of using *Drosophila* as a simple *in vivo* approach to investigate conserved neurobiological processes.

The use of fruit flies to model and investigate traumatic brain injury (TBI) was pioneered by Wasserman’s group. To elicit traumatic injury, Katzenberger et al. (2013) developed a “high-impact trauma” (HIT) device (Katzenberger et al., 2013, 2015a), which features a metal spring-loaded fly vial that can swing to introduce hits to the flies in the vial (Figure 1B). This first report provided early characterization of fruit flies subjected to mechanical trauma, including mortality/lifespan effects, climbing deficits, neurodegeneration, and immune responses secondary to trauma exposure. It also took advantage of the high-throughput nature of *Drosophila* by performing a genetic screen to compare mortality outcomes in mutant lines for innate immunity. Loss of highwire, a protein involved in Wallerian degeneration, which was first studied within the fly, is protective against cell death and degeneration following head injury exposure (Hill et al., 2020). Additional *Drosophila* TBI models have been developed, including those that can deliver head-specific impacts (Barekat et al., 2016; Sun and Chen, 2017; Saikumar et al., 2020; Behnke et al., 2021; Saikumar et al., 2021; van Alphen et al., 2022; Figure 1B). A summary of different *Drosophila* TBI models and the key findings from research using these models is provided in Supplementary Table 1. These models have also been extensively covered by other reviews (Shah et al., 2019; Buhlman et al., 2021; Aggarwal et al., 2022). It should be noted that studies using *Drosophila* TBI models have recapitulated several key aspects of trauma found in humans and other preclinical models of trauma, including acute stress responses such as oxidative stress and lysosomal activity, progressive neurodegeneration, neuronal hyperexcitability, and glial-specific responses (Barekat et al., 2016; Sun and Chen, 2017; Saikumar et al., 2020; Behnke et al., 2021; Saikumar et al., 2021; van Alphen et al., 2022). The short lifespan of fruit flies also enables the lifelong monitoring of brain deficits elicited by early exposure to mild head traumatic impacts (Behnke et al., 2021). The findings that female adult flies exhibit higher elevations of neuronal activity and more severe brain deficits later in life than male flies indicate the existence of sex differences in age-dependent development of neurodegenerative conditions after mild TBI exposure (Behnke et al., 2021). Below we will discuss how *Drosophila* may be utilized to investigate aging effects and sex differences in late-life emergence of neurodegenerative conditions after mild head injuries.

## Use of *Drosophila* melanogaster to understand aging effects

Aging usually refers to getting chronologically older, whereas normal aging or senescence is usually defined by the changes in physiology that occur naturally over time. *Drosophila* is a common model organism used in the study of aging (López-Otín et al., 2013). In 1916, Jacques Loeb and J. H. Northrop discovered that different ambient temperature can drastically alter *Drosophila* lifespan (Loeb and Northrop, 1916), and established that a 10°C reduction in temperature resulted in an approximate doubling of lifespan. This gave rise to the rate-of-living theory (Pearl, 1928), which suggests that an organism’s lifespan may be determined by its rate of living, in other words the rate of energy expenditure or metabolism. In the next hundred years or so, research using *Drosophila* and other model organisms painted a more detailed picture of aging; aging results from a combination of interconnected mechanisms and interactions between different loss-of-function phenotypes. Genetics have a significant impact on the longevity of animals (Finch and Ruvkun, 2001), but non-genetic factors such as nutrition, environment, and lifestyle also play an important role. *Drosophila* is a great model organism for the study of both genetic and non-genetic factors that contribute to aging.

Many aspects of the genetic basis to aging are conserved across many species (Piper and Partridge, 2018). The expression patterns of over 20% of genes in the fly genome change with age (Pletcher et al., 2002; Davie et al., 2018). During aging, RNA content and cell size decrease drastically but cellular identity is unaffected in old brains (Lints et al., 1984; Le Bourg, 1987). There is an increase in glia, which is also seen in human aging (Soreq et al., 2017). Mild dietary restriction is found to extend lifespan in yeast, worms, flies, rats, and monkeys (Colman et al., 2009). It is believed that nutrient sensors and their downstream signaling pathways mediate these changes. Furthermore, the fly gut is also believed to affect fly lifespan as it is both important for nutrient absorption and defense against harmful microbes and toxins. As flies age, their gut becomes more prone to dysplasia and over-proliferation. Differentiation of stem cells is more likely to go wrong in old age, and more so in female flies (Wang and Jones, 2011). However, dietary restriction seems to have no substantive effects on senescence of behaviors such as olfactory avoidance (Bhandari et al., 2007). Additionally, there is a plethora of research highlighting other conserved metabolic processes and signaling pathways in aging, such as the insulin or insulin growth factor signaling pathway (Chen et al., 1996; Brogiolo et al., 2001; Tatar et al., 2001; Broughton et al., 2005; Toivonen and Partridge, 2009), genes involving oxidative stress (Liguori et al., 2018), and histone deacetylases (Yu et al., 2021), all of which can influence longevity and senescence. This suggests that aging is an evolutionarily conserved process and at the very least, some basic biological mechanisms that regulate aging are conserved between humans and flies.

**Figure 2:**
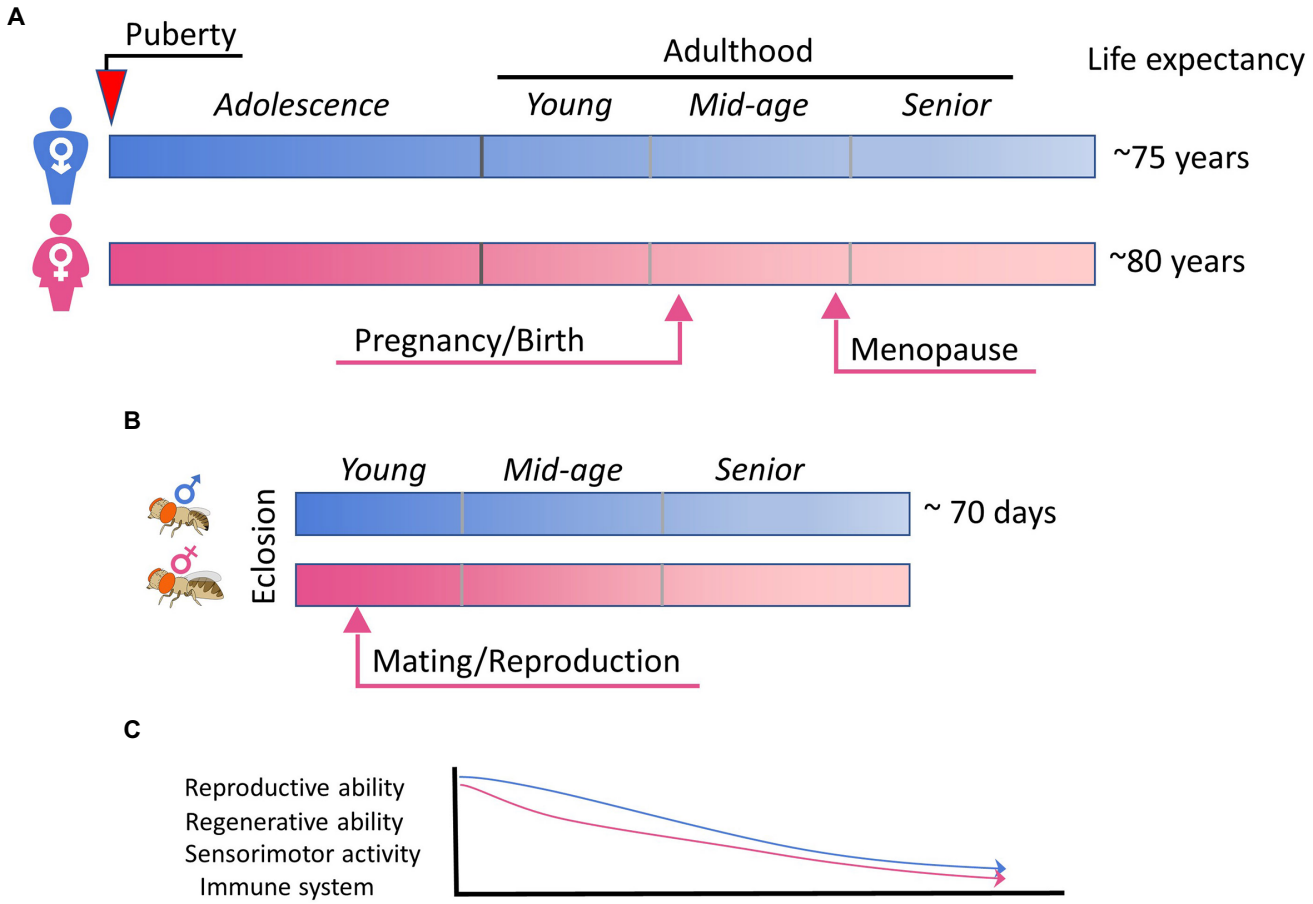
Aging and sex differences in human and fly. **(A)** A simplified summary of sex maturation, reproduction, and aging in human. Key events leading to physical, physiological, and hormonal changes in male and female are indicated arrows. **(B)** The schematic summary of the short lifespan of *Drosophila* flies, in which mating is known to trigger extensive changes in females. **(C)** A schematic plot highlighting the aging-associated functional declines that are common for human and fly, sexually dimorphic, and influenced by sex hormones and/or reproductive status.

Flies exhibit aging behavior comparable to human aging, such as the decline of locomotor activity, cognition, sensory responses, sleep, reproductive behavior, and the immune system (Iliadi and Boulianne, 2010) but over a much shorter time frame than human (Figure 2). Healthy fruit flies raised at 25°C have a median lifespan of 70–80 days and a maximum of around 100 days depending on the genetic background. As flies age, their exploratory locomotor activity (Le Bourg, 1983) and negative geotaxis behavior (Grotewiel et al., 2005) gradually decrease. However, there seems to be no significant correlation between the amount a fly moves and their eventual lifespan (Lints et al., 1984; Le Bourg, 1987). There are significant sex differences in aging-related changes to locomotor activity; female motor activity declines earlier in life than male (Lints et al., 1984). Aging also affects sensory responses and learning behaviors such as those seen in olfactory avoidance in flies (Tamura et al., 2003). Additionally, circadian rhythm changes accompany aging in flies. The decrease in circadian rhythm intensity and fragmentation in sleep–wake patterns is conserved across different species (Koh et al., 2006). Altering sleep can decrease lifespan and spur senescence (Bushey et al., 2010). Sexual reproductive behavior is also closely tied with aging, where aging is generally associated with decrease in reproductive behavior and fecundity (Toivonen and Partridge, 2009). Increasing sexual activity decreases lifespan in both female (Partridge et al., 1987) and male flies (Partridge and Farquhar, 1981), whereas decreasing reproductive behavior can increase lifespan and resistance to environmental stress (Yamamoto et al., 2013). Finally, fly immune function shows age-related decline, like that seen in humans and other vertebrates. Unlike the vertebrate immune system, which consists of both innate and adaptive or acquired immunity (Vivier and Malissen, 2005), *Drosophila* only have innate immunity wherein pathogens are recognized using innate receptors that trigger activation of downstream signaling pathways and immune responses (Hoffmann, 2003). Aging eventually leads to a reduced ability to combat disease and infections. Interestingly, immune response genes are often found to be upregulated with age in *Drosophila* transcriptome studies (Pletcher et al., 2002; Zerofsky et al., 2005; Ramsden et al., 2008; Carlson et al., 2015; Kubiak and Tinsley, 2017), which results in a higher expression of antimicrobial peptides (AMPs). This age-related increase in AMP expression has been shown to be tightly linked to intestinal barrier dysfunction (Rera et al., 2012). The stronger net response to infections in older flies is assumed to stem from the fact that younger flies readily rid the body of the infection, whereas microbes in older flies live longer and thus continuously activate the immune system. In line with this, effects of chronic NF-κB signaling are associated with age-related neurodegeneration in the *Drosophila* brain and nervous system (Petersen and Wassarman, 2012), and interventions that reduce the age-associated dysregulation of NF-κB signaling extend lifespan (Guo et al., 2014). Interestingly, TBI in *Drosophila* has been shown to cause the acute upregulation of AMPs and activation of the NF-κB pathway (van Alphen et al., 2022), suggesting the involvement of the immune pathway in injury responses as well as intersection with the aging pathway. In humans, chronic inflammation is suggested to be one of the most important causes of post-traumatic neurodegeneration (Faden and Loane, 2015). Notably, there are also sex differences in immune senescence (Kubiak and Tinsley, 2017); in males this occurs principally due to age-related deterioration in barrier defenses, whereas in females there are less changes related to barrier defense and more related to decreases in innate immunity.

The advantages of using flies in aging research are obvious; most prominently, the fly’s short lifespan and genetic tractability. Disadvantages include difficulty in characterizing aging behaviors and the fact that flies do not have complex socioeconomical pressure. We also do not know what flies really die of (Piper and Partridge, 2018), though research in tissue and metabolic constraints are beginning to suggest some mechanisms. There are a handful of *Drosophila* studies of TBI that examine age as a risk factor for neurodegeneration that not only recapitulate injury-induced phenotypes but also reveal aging-related vulnerabilities. Studies using the HIT model (Katzenberger et al., 2013, 2015a) examined multiple age groups ranging from 0 to 28 days of age and found that age-associated processes lower the primary injury threshold of death within the first 24 h after injury. They also found that aging exacerbated injury-induced neurodegeneration in the flies that survived 14 days following injury. The piezo-electric actuator model (Saikumar et al., 2020, 2021) has been utilized to examine chronic time-points (up to 28 days after injury) in which chronic activation of the immune system promotes neurodegeneration after injury at a young age. In a similar vein, Behnke et al. (2021) examined the lifelong sensorimotor behaviors and brain structure after the flies receive mild head trauma at young age (3 day), and found that immediate elevation of neuronal activity contributes to chronic neurodegeneration. Future studies may examine intersections of mechanisms of aging and mTBI to better understand the molecular networks that connect aging and accelerated progression of long-term brain deficits after mild head trauma.

## Sex dimorphism in *Drosophila* and neurodegeneration

Fruit flies are morphologically characterized as either male or female. In similar fashion to humans, sex determination in *Drosophila* is governed by the number of X chromosomes in the fertilized egg (Bridges, 1916). Two X chromosomes gives rise to a female fly, whereas XY gives rise to a male fly. The downstream pathways that determine sex dimorphism differ quite a bit between human and fly (Heller, 2010), though there are remarkable parallels when considering sex-specific variables such as the innate immune system and reproduction. Here, we will only discuss *Drosophila* pathways or mechanisms like those in humans which may contribute to sex differences in vulnerability to neurodegeneration.

In our previous discussion of possible sex-related mechanisms that affect after injury response, we highlighted the immune system as a mediator of later onset of neurodegeneration and a source of sex difference. In flies, sex differences exist in the immune system. Many of the genes in the innate immune pathways are found on the X chromosome and exhibit sex-specific induction following infection (Taylor and Kimbrell, 2007; Hill-Burns and Clark, 2009) as in the activation of Toll and immune deficiency signaling. Both of these signaling pathways are highly homologous to mammalian immune pathways (Kimbrell and Beutler, 2001). Though flies lack microglia, they do have ensheathing glia which are responsible for engulfing cellular debris following injury (Doherty et al., 2009). Unlike humans and other mammals, *Drosophila* do not have sex gonadal hormones such as estrogen, progesterone, and testosterone associated with sexual maturation and hormonal cycles associated with reproductive status. It is currently unclear whether sex-specific hormones in the fly, i.e., ecdysone, affect the immune system in the adult fly, though ecdysone is known to affect immunity during development (Meister and Richards, 1996; Flatt et al., 2008; Rus et al., 2013; Tan et al., 2014). Additionally, the fly immune system is known to be activated by TBI, though findings only pertain to male flies (Katzenberger et al., 2015b; Byrns et al., 2021). Sex differences in the immune response warrant the inclusion of female flies in the investigation of immune contributions to TBI-induced neurodegeneration.

Reproductive status is an important factor for sex differences in neurodegeneration due to remarkable changes in metabolism, fluctuations in female sex hormones, and alterations in the immune system. Here we discuss the fly post-mating response and its role in affecting the sexually dimorphic injury response. *Drosophila* are considered adults and are fertile within hours after eclosion. In humans, the reproductive system fully matures during puberty. In the female fly, mating elicits two types of major changes: increase in egg-laying and reduction in mating receptivity (Liu and Kubli, 2003). Other components of the post-mating response include changes in metabolism (increased intake of food (Carvalho et al., 2006), changes in food preferences for yeast and salt (Ribeiro and Dickson, 2010; Vargas et al., 2010; Walker et al., 2015), decreased intestinal transit (Cognigni et al., 2011; Apger-McGlaughon and Wolfner, 2013), changes in sleep–wake cycles (Isaac et al., 2010; Garbe et al., 2016; Dove et al., 2017) and altered immune system (Peng et al., 2005a; Short and Lazzaro, 2010; Short et al., 2012; Schwenke and Lazzaro, 2017). Some of these changes are mediated by ecdysone, a fly hormone structurally similar to estrogen in humans (Mangelsdorf et al., 1995). The seminal protein sex peptide (SP) from male flies plays a crucial role in eliciting the post-mating response in female flies. SP is an accessory fluid protein (Acps) produced by male flies in their accessory glands (Colucci et al., 2006; Jang et al., 2018) and received by female flies during copulation. A sustained post-mating response requires SP (Peng et al., 2005a,b; Apger-McGlaughon and Wolfner, 2013; Avila et al., 2015), and SP binding to its receptor is responsible for the switch in mating status in females. Overall, post-mating responses are considered evolutionarily beneficial as they maximize reproduction, though that may come with a cost; mating with males or just continuous exposure to male flies can significantly reduce lifespan in female fruit flies (Partridge et al., 1987; Fowler and Partridge, 1989). In humans, pregnancy elicits drastic changes to the body. These include changes in metabolism (Robinett et al., 2010), immune system (Aghaeepour et al., 2017), neurobiology (Hoekzema et al., 2017), and hormone fluctuations (Brunton and Russell, 2010), which are vital for the maintenance of pregnancy. Other sex-related changes in a healthy female’s life such as puberty, menstruation, or menopause elicit changes on a much smaller scale when compared to pregnancy (Cognigni et al., 2011; Apger-McGlaughon and Wolfner, 2013). A higher number of pregnancies is believed to be linked to elevated risk of AD related dementia (Isaac et al., 2010; Garbe et al., 2016; Dove et al., 2017), suggesting that factors associated with reproductive status, such as higher levels of female sex hormones, could play a role in AD neurodegenerative pathology. Some other studies show contradictory findings, where pregnancy and reproduction can be neuroprotective (Fox et al., 2018). This highlights the need for animal studies with better controls of hormonal cycles and increased sampling points.

Metabolic changes are known to affect neurodegeneration (Muddapu et al., 2020). In all animals, the production of progeny requires a significant energy investment. Metabolic changes during pregnancy are important for ensuring a healthy development and delivery of offspring. Not only does the need for substrates such as glucose, lipids, and proteins increase, the requirement for water, iron and calcium also change (Soma-Pillay et al., 2016). Similarly, female flies alter aspects of nutrient intake and digestion to meet the energy demands of egg production and maintain energy homeostasis. Not only does food intake increase by almost double (Carvalho et al., 2006), but food preferences are also shifted towards salt (Walker et al., 2015) and yeast (Ribeiro and Dickson, 2010; Vargas et al., 2010; Walker et al., 2015), which increases reproductive output. It is likely that changes in metabolism are only one of several changes induced by reproduction that may affect the fly’s vulnerability to developing neurodegeneration.

Along with metabolic changes, the female fly also experiences hormonal alterations after mating, which may affect neurodegeneration. Sex peptide is thought to induce release of steroid hormones, including Juvenile Hormone (JH) and ecdysone (20HE), due to the increase of 20HE titers in ovaries and a similar increase in the hemolymph of mated flies (Ameku and Niwa, 2016). Though the role of steroid hormones in developmental stages is better understood (Ameku and Niwa, 2016; Ahmed et al., 2020; White et al., 2021) than their role in reproduction, we now know that ecdysone produced by the ovaries is required for female fertility (Garen et al., 1977). Additionally, SP elicits steroid signaling from the ovaries to the gut to promote enlargement of the abdomen (Ameku and Niwa, 2016; Ahmed et al., 2020; White et al., 2021) and intestinal stem cell proliferation (Ahmed et al., 2020; Zipper et al., 2020). Although these transformations augment fecundity of the female fly by increasing energy uptake, they also increase female susceptibility to age-dependent tumors and thus potentially affecting overall health and reducing lifespan (Regan et al., 2016). In a similar vein, several studies in middle and older-age women suggest that high levels of overall hormone exposure accelerate brain aging and atrophy (Resnick et al., 2009; Kantarci et al., 2016; de Lange et al., 2020). However, there are other studies that suggest a protective role of female sex hormones (Ha et al., 2007; Song et al., 2020). The mechanisms underlying the effects of sex hormones on neurodegeneration are currently unclear, but future work using *Drosophila* and other animal models may help dissect this complex issue.

How does the sexually dimorphic immune system affect neurodegeneration? Changes in the immune system are known to alter trajectories of neurodegenerative disease such as CTE (Mason and McGavern, 2022). Sex differences in the immune system is evolutionarily conserved across many species and is strongly affected by age (Klein and Flanagan, 2016). Besides genetic factors, environmental factors and hormone cycles can also contribute to varying immune regulation pathways between the two sexes. In both flies and humans, immune responses are typically higher in females than in males (Klein and Flanagan, 2016). Within the female sex, reproductive status can drastically alter the immune system. In female flies, mating and exposure to SP reliably induces changes of the innate immune defense system (Short and Lazzaro, 2010; Short et al., 2012; Schwenke and Lazzaro, 2017). Female flies suffer a reduced ability to defend against certain bacterial pathogens after mating (Short and Lazzaro, 2010). Interestingly, when compared to virgins and females mated to sex peptide-less and sperm-less males, mated females exhibit lower survival rate and AMP expression. Females that fail to produce eggs demonstrate no effect of mating on immune defense (Short et al., 2012). This process has been shown to be mediated by steroid hormones. In virgin females, application of JH can phenocopy the immunosuppression observed in mated females while ablating JH and its downstream receptors induces virgin levels of resistance to bacterial infection (Schwenke and Lazzaro, 2017). This reproduction/ immune system tradeoff is seen also in humans (Westendorp et al., 2001; Abu-Raya et al., 2020). Notably, the risk and severity of certain infections such as urinary tract infections (Schnarr and Smaill, 2008) and pneumonia (Sheffield and Cunningham, 2009) are increased. This suggests that like in the fly, some energy from maintaining the mother’s immune system may be diverted to maintaining pregnancy and health of the fetus.

Finally, reproduction also alters plasticity at the synaptic level. The reproductive status of the female fly affects their aversive long-term memory, suggesting long-lasting changes in the function of specific neurons with homologous modulatory functions to the hypothalamus (Scheunemann et al., 2019). Similarly, human pregnancy is associated with various changes to functional and structural plasticity such as increase in neurogenesis, remodeling of synaptic morphology, and alterations in connectivity (Hoekzema et al., 2017; Barba-Müller et al., 2019; Hoekzema et al., 2022). The impairment of synaptic plasticity has been implicated in the development of neurodegenerative diseases such as AD (Sheng et al., 2012). Therefore, it is likely that reproduction-related synaptic changes contribute to female vulnerability to developing neurodegenerative conditions.

Sex differences, particularly those related to reproduction, can affect response to neurodegenerative stimuli such as mTBI. In both fly and humans, we expect changes in metabolism, hormone levels, and immune responses to affect neurodegeneration after mTBI, though the specific mechanisms remain to be elucidated. To date, *Drosophila* studies overwhelmingly only use male flies or do not consider reproductive status. In a severe TBI model, the HIT model, no sex differences have been found in terms of survival (Katzenberger et al., 2015a). However, female flies show more gene transcript changes than males, particularly immune response genes and mitochondrial genes (Shah et al., 2020). The same research group also separately found sex differences in survival following injury in tau knock-out flies, though it appears that tau is not involved (Shah et al., 2021). In congruence, Behnke et al. found that behavior dysfunction and pathology after mild injury was more pronounced in female flies than male flies when injured at 3–5 days of age (Behnke et al., 2021). Therefore, there is a great potential in using the fly model to study sex as a contributing factor to neurodegenerative mechanisms. Advantages of using flies include short life cycles and easy manipulation of the reproductive process using available genetic tools. Disadvantages include some disparities between the species in terms of sex differences, including a limited number of *Drosophila* orthologs of human hormonal genes.

## Summary and future studies

The complex nature of the inquiry into neurodegenerative diseases requires a variety of animal models and innovative strategies. Studying mTBI-induced neurodegeneration is additionally challenging because even though animals or human patients can recover from acute concussive symptoms in the short term, other symptoms such as impaired cognition, depression, and dementia can appear much later in life. To gain mechanistic understanding of disease onset, progression, and contributing factors, one must venture beyond population observations and into cellular and molecular manipulations. Longitudinal human and animal studies that follow the entire disease progression may be several years to several decades in length and can be very costly and difficult to carry out, which contributes to the current dearth of mechanistic findings. When compared with other animal models, *Drosophila* possess unique advantages such as a short lifespan, simple but conserved nervous system, a variety of genetic tools that allows for target-specific manipulation and examination, and ease and affordability of care. In this review, we have highlighted several *Drosophila* mTBI models, which provide the groundwork from which mechanistic insights can be obtained. Several of these models inflict mild, non-invasive, and headfirst injuries that have been shown to elicit neurodegeneration later in life, phenocopying observations from human populations. Future work using these models or modifications of these models can fill in the gaps in our understanding of neurodegeneration caused by exposure to mTBI. Finally, we highlight the potential for *Drosophila* models to be utilized in investigating age and sex as contributing factors to mTBI-induced neurodegeneration. Vulnerable populations like the aging population and females may require different treatment and care after mTBI exposure. *Drosophila* mechanisms of aging are highly conserved with humans; therefore, it is highly likely that the mechanisms related to aging-induced vulnerability to neurodegeneration are also conserved. Furthermore, though *Drosophila* and human sexual dimorphism are very different, the two species share remarkable similarities in reproductive pathways, which are suggested to be implicated in neurodegeneration following mTBI. Further investigation of female vulnerability is warranted. Using available *Drosophila* models, we can identify possible therapeutic targets that are tailored for populations that are at higher risk of developing neurodegeneration. Once possible therapeutic targets are found, *Drosophila* also allows for high throughput genetic screening and drug screening before moving on to higher-order preclinical and clinical studies.

## Author contributions

CY led the team effort in composing and completing this review article. CY and JB contributed a substantial amount of writing. KH and JZ contributed to the writing and editing. All authors contributed to the article and approved the submitted version.

## Funding

This work is supported in part by research grants from National Institutes of Health to JZ (GM083889 and MH129019), a Pilot grant to JZ from the NIH-funded Emory Specialized Center of Research Excellence in Sex Differences (U54AG062334), a Diana Jacobs Kalman Scholarship from American Federation for Aging Research (AFAR) to CY, and a T32 training grant from National Institute of General Medicine to Emory BCDB program (1T32GM135060-01).

## Conflict of interest

The authors declare that the research was conducted in the absence of any commercial or financial relationships that could be construed as a potential conflict of interest.

## Publisher’s note

All claims expressed in this article are solely those of the authors and do not necessarily represent those of their affiliated organizations, or those of the publisher, the editors and the reviewers. Any product that may be evaluated in this article, or claim that may be made by its manufacturer, is not guaranteed or endorsed by the publisher.

## Supplementary material

The Supplementary material for this article can be found online at: https://www.frontiersin.org/articles/10.3389/fnins.2023.1150694/full#supplementary-material
