# Supplementary material for: Drosophila melanogaster as a model to study age and sex differences in brain injury and neurodegeneration after mild head trauma
Source: Front Neurosci. 2023 Apr 3;17:1150694. doi: 10.3389/fnins.2023.1150694 (PMC10106652; doi:10.3389/fnins.2023.1150694)
Supplement: Supplementary file 1 [file Table_1.docx]

**TABLE: *Drosophila* Models for TBI-related Research**

| Model | Head-specific injury | Throughput potential | CO_2_ use for injury induction | Experimental design | Main findings |
| --- | --- | --- | --- | --- | --- |
| High-Impact Trauma (HIT) device ^1,2^ | no | High | no | - Used multiple age groups to characterize acute mortality, long-term survival and climbing deficits, as well as neuropathological evaluation of vacuoles to assess neurodegeneration - Used both male and female flies - Delivered repetitive injuries with lethal potential | - Single and repetitive injuries elicit acute mortality within 24h (~5% mortality following single exposure and ~20% following 4 repetitive exposures) - Acute activation of innate immune response seen following injury - Injury exacerbated age-related neurodegeneration seen 14d post-injury - performed high-throughput genetic screen to compare mortality outcomes in mutant lines for innate immunity |
| Tissue Homogenizer model ^3^ | no | Low | yes | - 7d old male or female flies received multiple injuries; different sexes were used for different assays and not used for comparative analyses | - Flies receiving multiple sublethal injuries showed increases in innate immunity and autophagy within 24h until 1 week following injury - Injured flies also exhibited acute climbing deficits, long-term circadian rhythm abnormalities and decreased overall survival |
| CO_2_ powered impactor model ^4^ | yes | Low | yes | - 2d old female flies received 1 or more head injuries from 4 different severities, including acutely sublethal (100% survival within 24h) to highly lethal (<10% survival) | - Following a single sublethal injury, flies exhibited a decreased locomotive response for 2d post-injury - Following repetitive sublethal injuries, flies exhibited a persistent locomotive deficient through at least 20d post injury and a decreased overall survival |
| *Drosophila* Closed Head Injury (dCHI) ^5^ | No | Low | No | - 3-7d old male flies received either 1, 5, or 10 injuries inflicted by the pin of a pull-type solenoid - Behavioral, pathological, and transcriptional analyses up to 7 days post injury. | - Injured flies exhibit immediate dose-dependent motor deficits, apoptotic cell death, fragmentation of sleep and reduction of lifespan. - Acute activation of the innate immune system (increase of AMPs up to 3d post injury) but returns to baseline by 7d. |
| *Drosophila* TBI (dTBI) or piezo-electric actuator model ^6-8^ | yes | High | yes | - 3d old males received a single injury of one of three injury severities - Behavioral and histological analysis was chronologically performed through 10d post-injury following severe injuries - Transcriptional changes measured until 28d post-injury | - Dose dependent deficits in locomotion, reduced acute and chronic survival and vacuole formation following severe injury - Recapitulated glial cellular reactivity following trauma - Measured transcriptional changes following injury, including glial AP1 which has a biphasic role in tau pathology following injury |
| HIFLI model ^9^ | yes | High-throughput | no | - 2-5d old male and female flies received multiple mild head injuries and were assessed for behavioral and histological examination throughout lifespan | - Female flies demonstrate worse persistent locomotive deficits following repetitive injury - Early injury-induced neuronal activity potentiates chronic neurodegeneration |

References cited:

1 Katzenberger, R. J. *et al.* A Method to Inflict Closed Head Traumatic Brain Injury in Drosophila. *Journal of visualized experiments : JoVE*, e52905, doi:10.3791/52905 (2015).

2 Katzenberger, R. J. *et al.* A Drosophila model of closed head traumatic brain injury. *Proceedings of the National Academy of Sciences* **110**, E4152-E4159 (2013).

3 Barekat, A. *et al.* Using Drosophila as an integrated model to study mild repetitive traumatic brain injury. *Scientific reports* **6**, 25252 (2016).

4 Sun, M. & Chen, L. L. A Novel Method to Model Chronic Traumatic Encephalopathy in Drosophila. *Journal of visualized experiments: JoVE* (2017).

5 van Alphen, B. *et al.* Glial immune-related pathways mediate effects of closed head traumatic brain injury on behavior and lethality in Drosophila. *PLoS Biol* **20**, e3001456, doi:10.1371/journal.pbio.3001456 (2022).

6 Byrns, C. N., Saikumar, J. & Bonini, N. M. Glial AP1 is activated with aging and accelerated by traumatic brain injury. *Nat Aging* **1**, 585-597, doi:10.1038/s43587-021-00072-0 (2021).

7 Saikumar, J. *et al.* Inducing different severities of traumatic brain injury in Drosophila using a piezoelectric actuator. *Nature Protocols*, 1-20 (2020).

8 Saikumar, J., Byrns, C. N., Hemphill, M., Meaney, D. F. & Bonini, N. M. Dynamic neural and glial responses of a head-specific model for traumatic brain injury in Drosophila. *Proc Natl Acad Sci U S A* **117**, 17269-17277, doi:10.1073/pnas.2003909117 (2020).

9 Behnke, J. A., Ye, C., Setty, A., Moberg, K. H. & Zheng, J. Q. Repetitive mild head trauma induces activity mediated lifelong brain deficits in a novel Drosophila model. *Sci Rep* **11**, 9738, doi:10.1038/s41598-021-89121-7 (2021).
